# Identification of the Single Immunodominant Region of the Native Human CC Chemokine Receptor 6 Recognized by Mouse Monoclonal Antibodies

**DOI:** 10.1371/journal.pone.0157740

**Published:** 2016-06-23

**Authors:** Karim Dorgham, Cécile Dejou, Christophe Piesse, Guy Gorochov, Jérôme Pène, Hans Yssel

**Affiliations:** 1 Sorbonne Universités, Inserm U1135, UPMC Univ Paris 06 UMRS 1135, Centre d’Immunologie et des Maladies Infectieuses (Cimi-Paris), F-75013, Paris, France; 2 Institut de Recherche en Cancérologie, F-34090, Montpellier, France; 3 Sorbonne Universités, UPMC Univ Paris 06, IBPS-FR 3631, Service de Synthèse Peptidique, F-75005, Paris, France; 4 AP-HP, Groupement Hospitalier Pitié-Salpêtrière, Département d’Immunologie, F-75013, Paris, France; 5 Institute for Regenerative Medicine and Biotherapy, Inserm U1183, F-34295, Montpellier, France; University of Massachusetts Medical School, UNITED STATES

## Abstract

Chemokines and their receptors play an important role in cell trafficking and recruitment. The CCR6 chemokine receptor, selectively expressed on leukocyte populations, has been shown to play a deleterious role in the pathogenesis of various chronic inflammatory diseases and, as such, may constitute a prime target in the development of immunotherapeutic treatment. However, to date no neutralizing mouse monoclonal antibodies (mAbs) specific for this chemokine receptor have been reported, whereas information on small molecules capable of interfering with the interaction of CCR6 and its ligands is scant. Here, we report the failure to generate neutralizing mouse mAbs specific for human (hu)CCR6. Immunization of mice with peptides mimicking extracellular domains, potentially involved in CCR6 function, failed to induce Abs reactive with the native receptor. Although the use of NIH-3T3 cells expressing huCCR6 resulted in the isolation of mAbs specific for this receptor, they were not able to block the interaction between huCCR6 and huCCL20. Investigation of the anti-huCCR6 mAbs generated in the present study, as well as those commercially available, show that all mAbs invariably recognize a unique, non-neutralizing, immunodominant region in the first part of its N-terminal domain. Together, these results indicate that the generation of potential neutralizing anti-huCCR6 mAbs in the mouse is unlikely to succeed and that alternative techniques, such as the use of other animal species for immunization, might constitute a better approach to generate such a potentially therapeutic tool for the treatment of inflammatory disease.

## Introduction

CCR6 (CD196) is a CC chemokine receptor, involved in host defense and inflammation, especially at epithelial surfaces, that has two specific ligands, the chemokine CCL20 and a non-chemokine ligand β-defensin-2, an anti-microbial peptide produced by epithelial cells that line various organs [1–9]. CCR6 is expressed at the cell surface of CD4^+^ interleukin-17 (IL-17)-, IL-22- and TNF-α-producing T lymphocytes, a population with strong pro-inflammatory properties referred to as Th17 cells [10–12], as well as all circulating, naive and memory, but not germinal center, B lymphocytes [13]. CCR6 is also expressed by IL-17 and IL-22-producing innate lymphoid cells [14] and by immature dendritic cells, although its expression on the latter cells is lost following their maturation [15]. There is compelling evidence from experimental mouse models, as well as from clinical studies in human, that the CCR6/CCL20/Th17 axis is involved in the pathogenesis of various chronic inflammatory and autoimmune diseases, which has been well documented for multiple sclerosis and rheumatoid arthritis. In particular, myelin-specific T cell infiltration in the brain was reported to positively correlate with the expression of CCL20 in the choroid plexus of humans with multiple sclerosis or mice with experimental autoimmune encephalitis [16]. Moreover, *Ccr6* deficient mice are resistant to the induction of disease which is not due to a defect in the differentiation of Th17 cells in brain-draining lymph-nodes after induction of experimental autoimmune encephalitis, but rather the consequence of the failure of these cells to migrate into the inflamed central nervous system [16, 17]. Similar results with respect to lymphocyte migration have been obtained in the SKG mouse model of spontaneous experimental arthritis in which a preferential recruitment of Th17 cells to inflamed, CCL20-expressing, synovial joints was observed that could be inhibited with a neutralizing anti-CCR6 antibody [18], whereas polymorphisms in the *CCR6* gene were reported to be associated with rheumatoid arthritis susceptibility [19, 20].

It is important to note that autoimmune, CCR6-expressing, B cells also play an important role in the pathology of both multiple sclerosis and rheumatoid arthritis. Current biotherapy, specifically targeting and depleting B cells from the circulation with the anti-CD20 mAb Rituximab^®^ was found to result in a reduction of inflammatory brain lesions and clinical relapses in patients with relapsing-remitting multiple sclerosis [21]. Moreover, treatment of patients with rheumatoid arthritis with Rituximab^®^ also leads to a significant improvement of their clinical signs (Review in [22]). As all functionally mature B cells, like Th17 cells, express CCR6 at their surface, they are likely to be responsive to the migration-inducing effects of CCL20, strongly suggesting that the deleterious effect of both cell types in the pathogenesis of in these chronic inflammatory diseases is linked to the capacity of this receptor and its ligand(s) to control lymphocyte migration into inflamed tissue.

Consistent with the molecular mechanisms underlying the involvement of chemokines and their receptors in lymphocyte trafficking [22], both CCL20 [23] and β-defensin-2 [24] were reported to induce a conformational modification of the integrin CD11a/CD18 (LFA-1) that, following its interaction with CD54 (ICAM-1) expressed by inflamed endothelium, results in the arrest of CCR6-expressing Th17 lymphocytes on the latter cells. Although similar *in vitro* data for human B cells are lacking, it is very probable that their trafficking into inflamed tissue, mediated by CCR6 is orchestrated by the same molecular mechanisms. This chemokine receptor is therefore likely to represent a possible therapeutic target for the treatment of certain chronic inflammatory diseases.

In the present study, we report the failure to generate neutralizing mouse mAbs specific for huCCR6. The results show that all such mAbs generated by immunization of mice with huCCR6-expressing cells are directed at a single, non-neutralizing, region of the N-terminal domain of the native huCCR6.

## Materials and Methods

### mAbs and reagents

The following anti-huCCR6 mAbs were used in the present study (S1 Table): clone G034E3 (Biolegend), MM0066-3L1 (Abcam), 53103 (R&D Systems) and 11A9 (BD Biosciences). Anti-huCCR7 clone 150503 (BD Biosciences) was used as irrelevant control antibody. The pcDNA3.1-CCR6 or pcDNA3.1-CCR2 vectors were obtained from the Missouri S&T cDNA Resource Center.

### Cell culture

CCR6^+^ and CCR6^-^ T cell lines and clones used in this study were generated as described [25]. T cells were cultured in a feeder cell mixture consisting of irradiated (45 Gy) allogeneic peripheral blood mononuclear cells, irradiated (60 Gy) JY cells and 0.1 μg/mL of PHA (Remel Europe Ltd) in 24-well culture plates, propagated with rIL-2, and were used in experiments between 10 and 14 days after the onset of each propagation, as described [26]. All cultures and experimental procedures were carried out in Yssel’s medium [27], supplemented with 1% human AB^+^ serum (Etablissement Français du Sang, Lyon, France). The CHO-K1 and NIH-3T3 cell lines were transfected with the pcDNA3.1-CCR6 or pcDNA3.1-CCR2 vectors, using JetPEI 9 transfection reagent (Polyplus-Transfection), as described [24]. All cell lines used in this study were free of Mycoplasma infection as checked by fluorescence microscopy [28].

### Peptide synthesis

The peptides were synthesized using solid-phase FastMoc chemistry procedure on an Applied Biosystems 433A automated peptide synthesizer. Resin and Fmoc-protected amino acids were purchased from Merck Chemicals (Novabiochem) and Iris Biotech GMBH, respectively, and solvents from Carlo Erba reagents. Peptide synthesis was carried out as previously described [29]. Fmoc-Cys(Trt)-wang resin LL (0.29 mmol/g), Fmoc-Tyr(OtBu)-wang resin (0.64 mmol/g) and Fmoc-Leu-wang resin LL (0.3 mmol/g) were used for the synthesis of the following peptides respectively: CSTFVFNQKYNTQGSDVC (l-PA), representing the second extracellular loop (ECL-2), as well as MSGESMNFSDVFDSSEDY (N1-18) and FVSVNTYYSVDSEMLLCSLQEVRQFSRL (N19-47) that represent residues 1–18 and 19–42 of the N-terminal part of the molecule, respectively.

The cyclic c-PA peptide was obtained by the formation of a disulfide bond between Cys-1 and Cys-18 of the purified l-PA by dissolving and stirring the peptide (2 mg/mL) in a dimethyl sulfoxide/water (1:2 vol/vol) solution for 24 h at room temperature. Both the linear and disulfide cyclized peptides were purified by high-performance liquid chromatography and their molecular masses were determined by MALDI TOF-MS. Mass spectra full scan analysis of the purified peptides showed a major peak at m/z = 2041.62 for the linear form (l-PA) and a major peak at m/z = 2039.08 for the cyclized form (c-PA). The observed difference in molecular weight points corresponds to the formation of a disulfide bridge between the N- and C-terminal cysteine residues.

For the immunization procedure, peptides were coupled to KLH using the Pierce Imject EDC mcKLH spin kit and the Imject Maleimide activated mcKLH spin kit for c-PA and linear N19-47 peptides respectively.

### Immunization procedures

**Fig 1:**
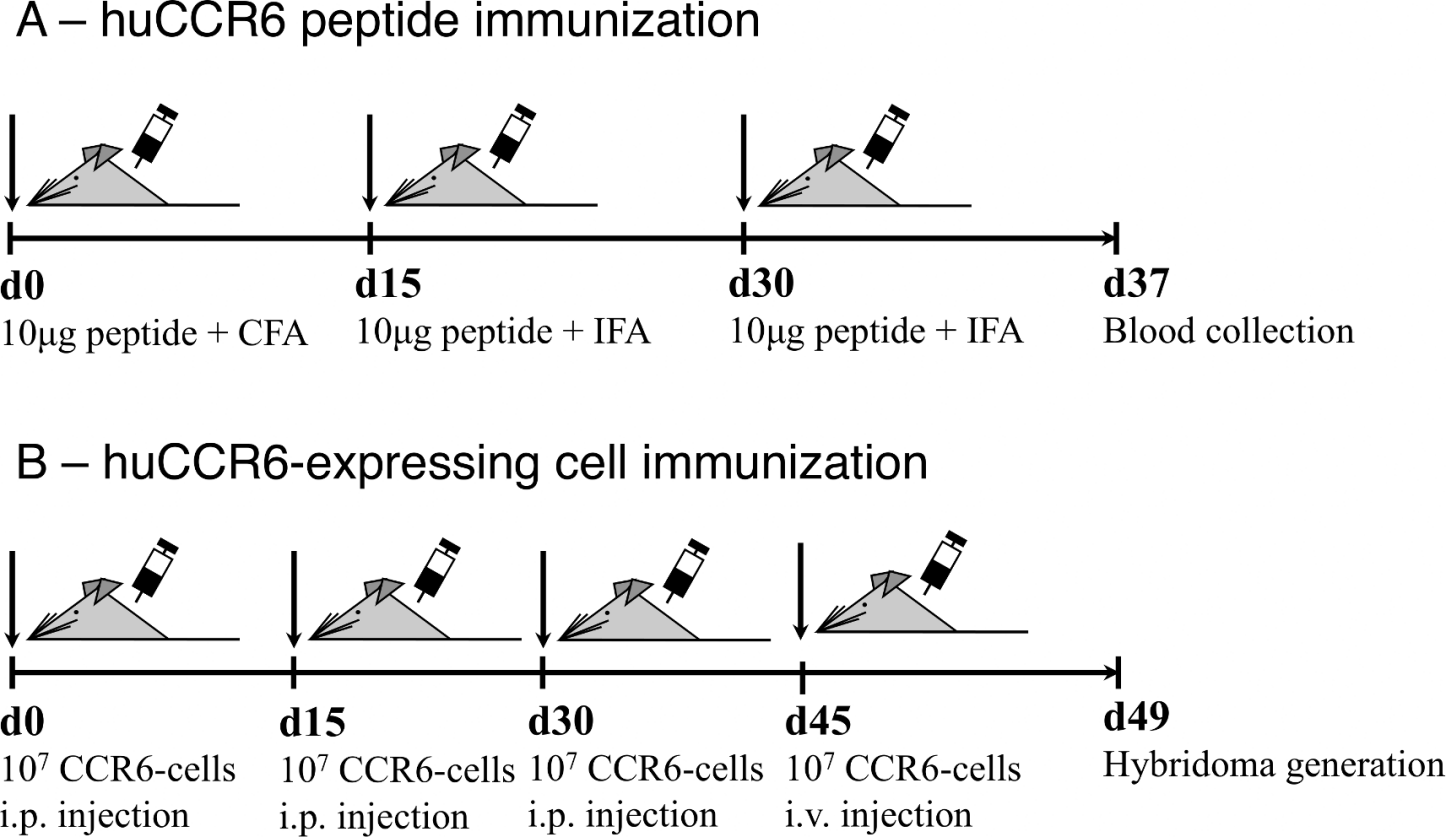
Immunization strategies to generate mouse anti-huCCR6 mAb. **(A)** Mice (n = 4) were injected three times i.p. at the indicated days with N19-47 or cPA peptides in the presence of Complete (CFA) or Incomplete (IFA) Freund’s Adjuvant. Serum was collected and the Ab specificity was determined at day 37 after the first immunization. **(B)** huCCR6-NIH-3T3 cells were used for two i.p. injections in mice (n = 4) at a 14 day interval and for a final i.v. boost injection at day 45, 4 days before generation of hybridoma cells.

The two immunization procedures are depicted in Fig 1. For peptide immunization, twelve-week-old female Balb/c mice (Janvier Laboratories) received three i.p. injections, at a 14 day interval, of 10 μg of peptide bound to KLH in Complete Freund’s Adjuvant (first injection) or Incomplete Freund’s Adjuvant (subsequent injections) (Fig 1A). Blood samples were collected retro-orbitally on the day of the third injection. Seven days after the last immunization, serum was collected by centrifugation and anti-peptide Ig titers were determined by ELISA (see below).

In an alternative immunization procedure, five-week-old Balb/c mice were injected i.p. three times with 10^7^ huCCR6-expressing NIH-3T3 cells, in the presence of Alum and CpG at 14 day intervals. For the generation of mAbs, mice received an i.v. boost injection with 10^7^ huCCR6-expressing NIH-3T3 (huCCR6-NIH-3T3) cells two weeks after the last immunization (Fig 1B). Four days later, the animals were euthanized, their spleen removed and a fusion between the homogenized splenocytes and X63/Ag8 plasmacytoma cells was performed using PEG 1500 (Roche Diagnostics). Hybridoma cells were selected with hypoxanthine, aminopterin and thymidine-containing medium (HAT media supplement, Sigma), culture supernatants were screened by flow cytometry as described below. Selected hybridomas were subcloned five times to obtain stable Ig-producing cells and maintained in culture, as described [30]. The experimental procedures were approved by the local ethical committee for animal experimentation in Montpellier, France (reference number CEEA-LR-12033).

### Identification of mAbs by flow cytometry

CCR6^+^ or CCR6^-^ CD4^+^ T cells, as well as CHO and NIH-3T3 cell lines, expressing huCCR6 or huCCR2, were used to screen sera from immunized mice and/or hybridoma culture supernatants for the presence of huCCR6-specific (m)Abs. One hundred thousand cells were incubated for 30 min with ten-fold steps serial dilutions of serum or undiluted culture supernatant, at 4°C, washed twice in PBS-2% FCS, followed by a 30 min incubation with a PE-conjugated goat anti-mouse Ig (BD Pharmingen) at the concentration of 10 μg/mL and were analyzed by flow cytometry using a FACSCalibur (BD Biosciences). A total of 10,000 events were acquired and data were analyzed with CellQuest software (BD Biosciences). Hybridomas producing the relevant mAb were stabilized by repeated subcloning, and mAbs were purified from culture supernatants using a protein G column by Proteogenix SAS.

### ELISA

Sera from immunized mice and purified mAbs were analyzed by ELISA as described [31]. Serially diluted serum or purified mAbs (at 1 μg/mL) in PBS-0.1% BSA-0.1% Tween20 were incubated for 2 h at room temperature in 96-well plates (Nunc-Immuno Plates Maxisorp), previously coated with 100 μL/well of peptide solutions at 1 μg/mL. The synthetic peptide derived from Tetanus toxin, (residues 830–843; Ctr-p: QYIKANSKFIGITE, Bachem), was used as an irrelevant peptide. After washing, bound IgG were revealed with 1/1,000 dilution of a horseradish-peroxidase (HRP)-conjugated goat anti-mouse IgG antibody (BD Biosciences).

### Molecular characterization of mAbs

RNA was extracted from 10^6^ hybridoma cells using Trizol reagent (Ambion, Lifetechnologies), according to manufacturer’s instructions. Reverse transcription—polymerase chain reaction (RT-PCR) and amplification of the IgG variable domains was performed as reported in Dorgham et al. [31], using oligonucleotide primers described for the first round PCR. DNA sequencing of PCR product was performed using an ABI Prism 3100 automatic sequencer (Applied Biosystems, Thermo Fisher Scientific) using the BigDye Terminator v3.1 Cycle Sequencing Kit and analyzed with the Sequencher® v4.7 sequence analysis software (Gene Codes Corporation). Identification of V, D, J genes and alleles of Ig was performed (S2 Table) with IMGT/V-QUEST program version: 3.3.5. [32, 33]

### Binding and internalization experiments

The binding titration of purified mAbs was carried out by flow cytometry in the presence of NaN_3_ to avoid internalization. Cells were incubated for 30 min with mAbs at 4°C in 100 μL of binding buffer (PBS, supplemented with 0.5% BSA and 0.1% NaN_3_) at various concentrations. Medium alone and isotype matched mAb were used as controls. For internalization assay, cells were incubated with mAbs for 1 hour at 37°C, 5% CO2, in supplemented culture medium. Cells were washed five times with cold binding buffer and stained for 30 min on ice with PE-conjugated goat anti-mouse Ig. Cells were washed twice and CCR6 expression was analyzed by flow cytometry on a FACSCanto with FACSDiva software (BD Biosciences).

### Fluorescence-based competitive binding assay

Competition of mAbs with huCCL20 for binding to huCCR6 was carried out using a competitive binding assay. One hundred thousand huCCR6-CHO cells were pre-incubated, either with mAb (up to 300 μg/mL) or huCCL20 (8 μg/mL), for 15 minutes at 4°C in 100 μL binding buffer. Alexa Fluor® 647-(AF647) conjugated huCCL20 (Almac) was added at a concentration of 80 ng/mL for 30 minutes at 4°C. Competition of huCCR6 with peptide for binding to mAbs was performed as follow: huCCR6-CHO cells were incubated at 4°C in 100 μL binding buffer with various concentrations of peptides and in the presence of 0.1 μg mAb. After washing, cells were stained with PE-conjugated goat anti-mouse Ig. Cells were washed three times with 1 mL of cold binding buffer and analyzed by flow cytometry, as described above.

### Calcium mobilization assay

Mobilization of Ca^2+^ from cytoplasmic stores was determined, as described previously [24] using huCCR6-CHO cells. A volume of 25 μL/well of mAb diluted in assay buffer was added on dye-loaded cells after 30 s of baseline acquisition to achieve the final indicated concentrations. Agonistic activity was monitored for 150 s using the Flexstation 3 fluorometer system (Molecular Devices) with excitation, emission and cut-off filter set to 485, 525 and 515 nm, respectively. To study the antagonist activity, recombinant huCCL20 (Peprotech) was added after 15 min incubation with the mAb in a volume of 25 μL/well to reach the EC80 (160 μg/mL) determined beforehand. Intracellular Ca^2+^ concentration was monitored for 150 s, as described above, and agonist and antagonist responses were determined, based on changes in fluorescence intensity over baseline (peak signal), as quantified using SoftMax Pro software.

## Results

### Immunization strategy to generate antibodies targeting the N-terminal part and the ECL-2 of huCCR6

Both the N-terminal region and the ECL-2 region of chemokine receptors have been identified as major binding domains for their chemokine ligands (review in [34]). Moreover, molecular characterization of CCR6 has confirmed the involvement of both regions in ligand binding, as well as receptor signaling and trafficking [35, 36].

Therefore, based on the structure of CCR5 [37], CXCR4 [38, 39] and CX3CL1 [40], as well as the reported capacity of cyclic ECL-2-derived peptides to induce the generation of CXCR4- [41] and CCR5-specific neutralizing antibodies [42], a set of huCCR6-specific peptides was designed and synthesized for the immunization procedure (Fig 1A).

**Fig 2:**
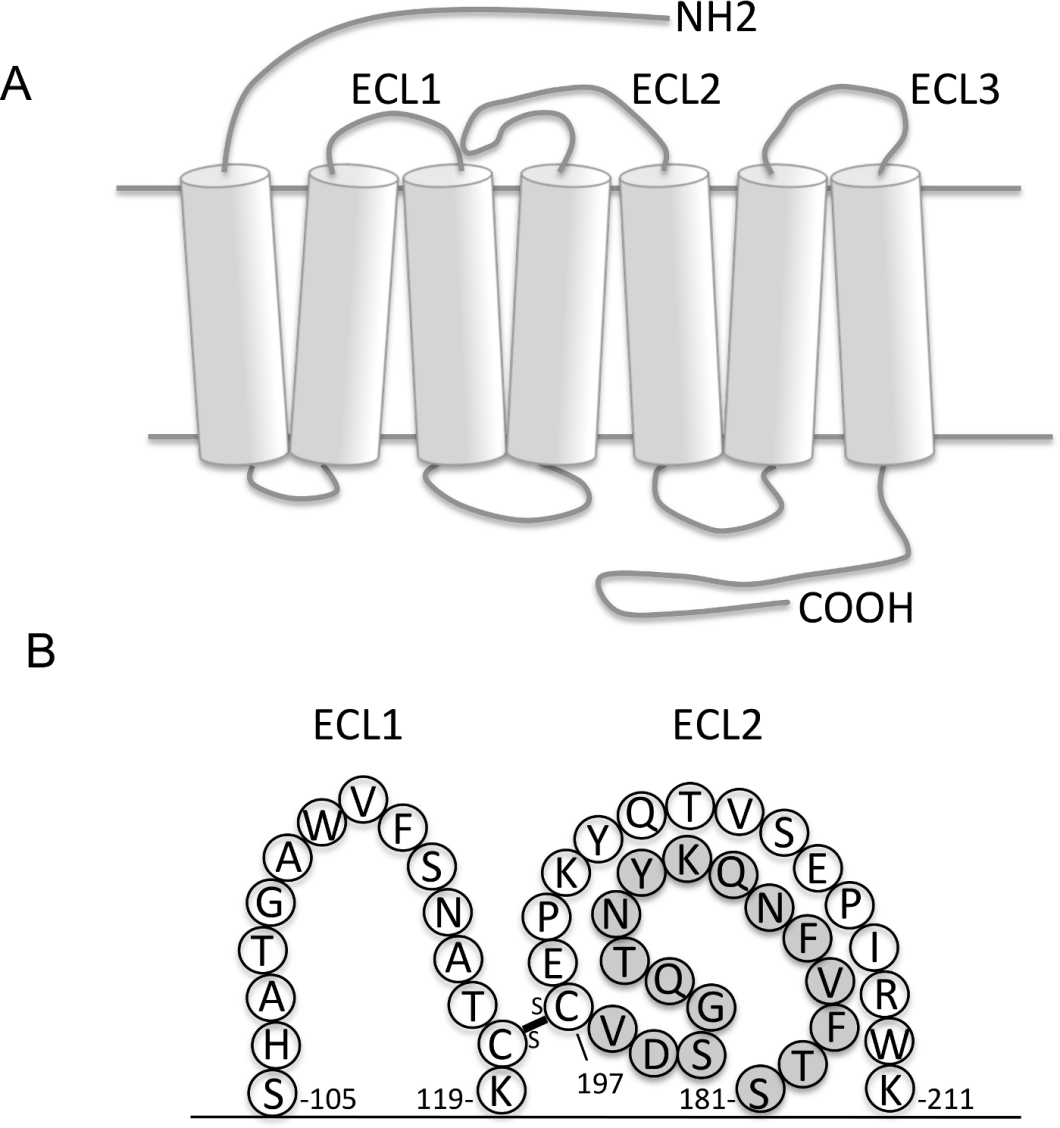
Predicted structure of huCCR6. **(A)** Representation of the serpentine structure of huCCR6 formed at the top by the extracellular domains consisting of the N-terminal end and three loops (ECL1-3), in the middle by the horizontal bands and cylinders, symbolizing the cell membrane and the seven transmembrane domains, respectively, and at the bottom by the intracellular domains consisting of three loops and the C-terminal end. **(B)** Representation of the peptidyl arch of the ECL2 of huCCR6 formed by the disulfide bridge between ECL-1 and ECL-2 (connecting line). Amino acids are listed with a single letter code. The horizontal line represents the cell membrane. The arch structure consisting of 16 amino acids (S181-V196, grey circles) was cyclized and used as immunogen.

The ECL-2 domain of huCCR6 is composed of 31 residues (S181 to K211) that create a 16 amino acid arch structure (S181-V196), containing a disulfide bond between C197 and the C118 residue of the ECL-1 (UniProtKB—P51684: Fig 2A and 2B). The corresponding peptide (linear Peptidyl Arch: l-PA = 181-STFVFNQKYNTQGSDV-196) was selected and cyclized (c-PA) by insertion of a cysteine at each extremity to mimic the native conformational region (Fig 2B).

Immunization of mice with KLH-conjugated c-PA induced high serum titers of peptide-specific antibodies in all animals, as demonstrated by specific ELISA (S1 Fig). Serum antibodies were found to bind both c-PA and I-PA, but not a control peptide. However, in flow cytometry experiments none of the sera recognized huCCR6 expressed by Th17 cells, indicating that the cyclic synthetic peptide used in the present study, does not seem to share an immunogenic region with the native ECL-2 motif of this chemokine receptor. Immunization of mice with this mimetic molecule is therefore unlikely to generate CCR6-specific mAbs and no fusions were carried out.

Immunization of mice with the first 18 residues of a huCCR6-derived synthetic peptide was reported to result in the generation of anti-huCCR6 mAbs, indicating that this part of the N-terminal portion (N1-18) of the chemokine receptor is immunogenic [43]. However, as these mAbs do not have neutralizing capacity a peptide immunization strategy was envisaged, using a synthetic peptide (N19-47) spanning the remainder of the N-terminal domain. Immunization with the N19-47 peptide also resulted in high, peptide-specific, serum titers (S1 Fig). However, no serum reactivity with either huCCR6-transfected cells or Th17 cells, could be detected by flow cytometry. Because of the total lack of reactivity of sera of the hyperimmune mice with native huCCR6, no fusions were carried out and an alternative strategy was devised.

### Immunization of mice with huCCR6-expressing NIH-3T3 cells

**Fig 3:**
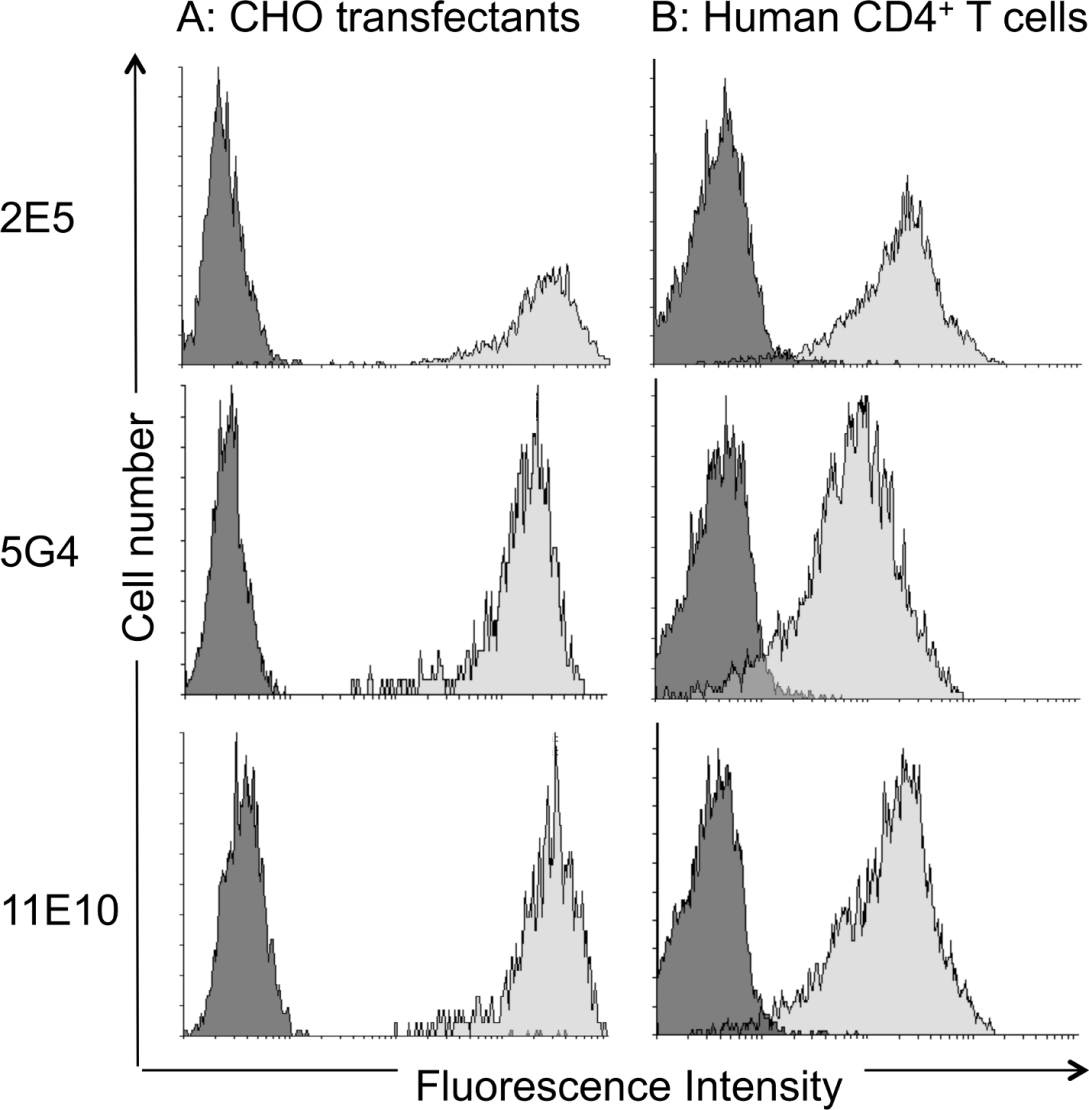
Specificity of mAbs generated by immunization with huCCR6-expressing cells. The specificity of the 2E5, 5G4 and 11E10 mAbs in the corresponding hybridoma supernatants was determined by flow cytometry, using **(A)** CHO-K1 cells, transfected with huCCR6 or huCCR2, respectively and **(B)** human CCR6^+^ and CCR6^-^ CD4^+^ T cells. The *x-axis* represents fluorescence (four-decade log scale), and the *y-axis* represents the relative cell number. Dark grey histograms from control cells are superimposed over light grey histograms of huCCR6^+^ cells. One representative analysis from four independent experiments is shown. The histogram overlay were performed using Flowing Software vs 2.5.

One approach to bypass incorrect presentation or weak expression of a complex epitope structure is to immunize either with the purified molecule or the receptor in the context of the cell on which it is expressed. Because of the particular nature of seven-transmembrane domain receptors and the stringent conditions required of its obtention in purified form, while maintaining its specific conformational structure, immunization with the biochemically purified or recombinant CCR6 protein is not feasible. Therefore, mice were immunized with huCCR6-NIH-3T3 cells resulting in the generation of high serum titers of Abs reactive with the latter cells, but not with wild type NIH-3T3 cells, as measured by flow cytometry, and a fusion was carried out. From a total of 2,000 hybridoma supernantants, three mAbs, 2E5, 5G4 and 11E10, were identified that recognized both huCCR6-CHO and native huCCR6-expressing Th17 cells, but neither pCDNA3.1-CCR2-transfected cells, nor huCCR6^-^ CD4^+^ T cells (Fig 3) and were further characterized. Both the 2E5 and 5G4 mAbs were of the IgG2a isotype and 11E10 was an IgG1 mAb, as determined by sandwich ELISA [44]. Sequence analysis of their variable domains revealed that the 2E5 and 5G4 clones were identical while 11E10 was a different mAb (S2 Table).

### Functional characterization of the generated anti-huCCR6 mAbs

**Fig 4:**
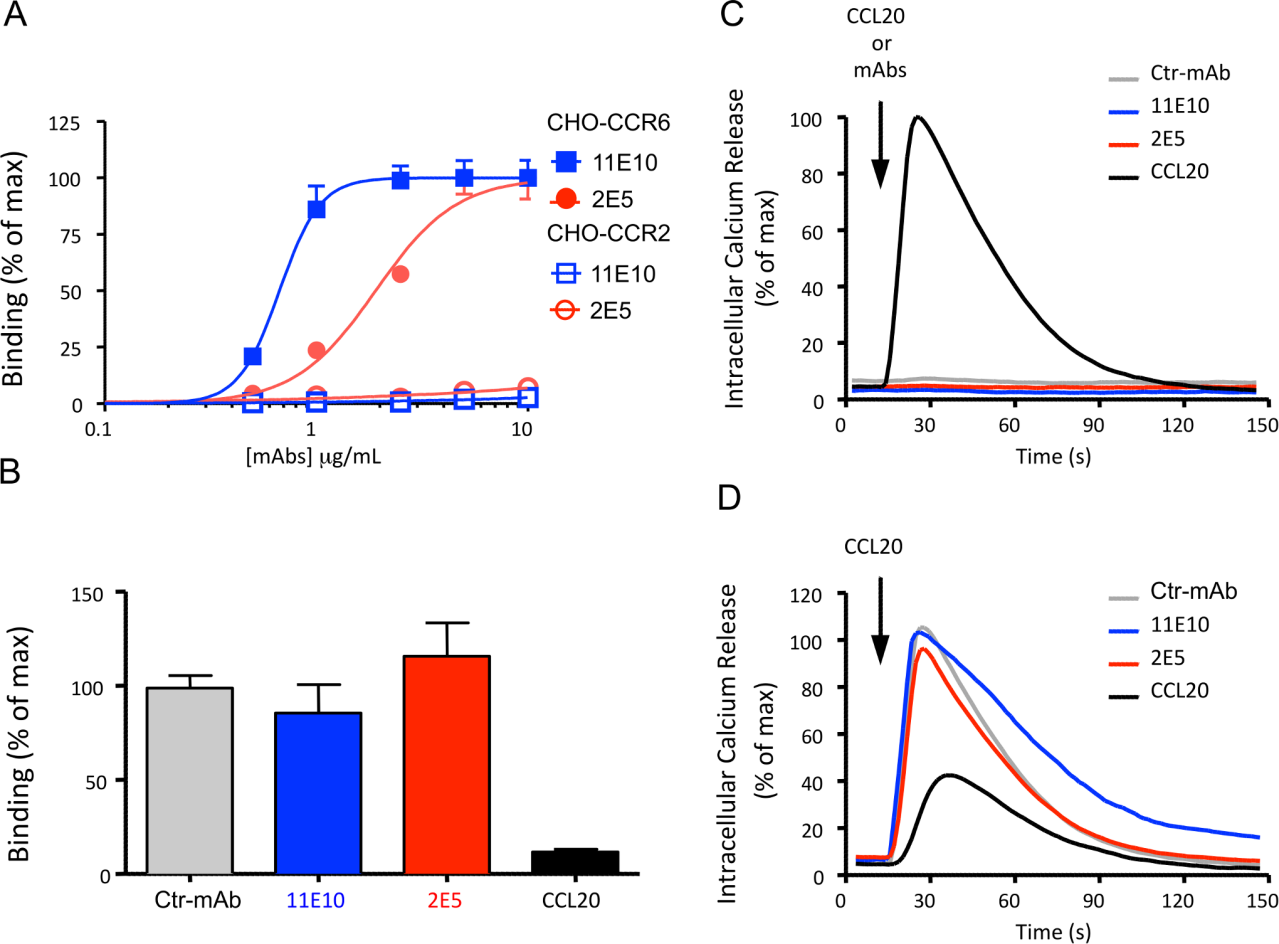
Functional characterization of anti-huCCR6 mAbs. **(A)** Binding titration of the 2E5 and 11E10 mAbs on huCCR6-CHO and huCCR2-CHO cells, analyzed by flow cytometry assay. After plotting the data (log(agonist) vs. normalized response, GraphPad Prism software), the EC50 was 1.911 μM for 2E5 and 0.6708 μM for 11E10. Mean values ± SEM of duplicate samples are shown in the graph. One representative analysis in duplicate from two independent experiments is shown. **(B)** Competitive binding of AF647-CCL20 (80 ng/mL) on huCCR6-CHO cells in presence of anti-CCR6 mAbs or a control mAb (Ctr-mAb) (300 μg/mL) or huCCL20 (8 μg/mL)). Maximum binding (without competitor) was used as 100% for normalization with GraphPad Prism software. Mean values ± SEM of pooled data from two independent experiments are shown in the graph. **(C,D)** Kinetics of calcium mobilization from internal stores in huCCR6-CHO cells following stimulation with **(C)** mAbs (100 μg/mL) or huCCL20 (80 ng/mL) and **(D)** inhibition of huCCL20-mediated Ca2+ flux (160 ng/mL) after an incubation for 15 min of the cells with mAbs (300 μg/mL) or huCCL20 (80 ng/mL). Maximum peak signal (without competitor) was used as 100% for normalization with GraphPad Prism software. One representative analysis of three independent experiments. Curves represent the mean values of triplicate measurements.

The efficacy of a mAb to exert functional activity on its specific receptor is determined by its affinity, as well as by its capacity to induce cell activation or to compete with the natural ligand for binding to this receptor. The binding capacity of 2E5 and 11E10 to huCCR6-expressing CHO cells was determined by flow cytometry analysis based on a binding titration of the mAbs at 4°C, in the presence of NaN_3_ to avoid receptor/antibody complex internalization. Maximal binding to huCCR6-CHO cells was observed at concentrations of 1 and 5 μg/mL for the 11E10 and 2E5 mAbs, respectively (Fig 4A). To perform receptor internalization analysis, the experiment was repeated by incubating the cells with the mAbs, in the absence of NaN_3_, at 37°C and 5% CO_2_. The binding titration curve was similar to that obtained at 4°C (S2 Fig), demonstrating that neither mAb triggers downmodulation of CCR6 expression from the surface of these cells.

To evaluate the capacity of 2E5 and 11E10 to compete with huCCL20 for binding to its receptor, huCCR6-CHO cells were incubated with various concentrations of the mAbs in the presence of 80 ng/mL of AF647-conjugated huCCL20. The fluorescence intensity, corresponding to maximum binding of huCCL20 in medium alone, was compared to that obtained in the presence of each mAb or a control mAb. Both 2E5 and 11E10 were unable to compete with AF647- conjugated huCCL20 for huCCR6 binding, as compared to the control mAb. Unlabeled huCCL20, at a concentration of 8 μg/mL, was used as a competitive positive control (Fig 4B).

Following its specific interaction with the molecule to which it has been raised, a mAb may exert agonistic or antagonistic activity or, alternatively, no functional activity at all. To discriminate between these possibilities, the capacity of the 2E5 and 11E10 mAbs to elicit mobilization of Ca^2+^ from internal stores or to inhibit huCCL20-mediated Ca^2+^ flux in CCR6-expressing cells was determined. The addition of 2E5 or 11E10, even at a concentration of 100 μg/mL, did not induce a Ca^2+^ flux in huCCR6-CHO cells (Fig 4C), showing that neither mAb has agonistic activity, contrary to huCCL20 that, at a concentration of 80 ng/mL, induced a strong Ca^2+^ response. The capacity of both mAbs to inhibit the huCCL20-induced Ca^2+^ flux was also determined at various concentrations. Pre-incubation of the cells with 80 ng/mL huCCL20 effectively reduced the peak fluorescence signal obtained in the absence of the ligand, demonstrating the desensitization of the receptor (Fig 4D). In contrast, the presence of either mAb, at a concentration as high as 300 μg/mL, did not affect the peak fluorescence signal which was comparable to that observed in the presence of a control mAb.

### Characterization of the immunogenic region of huCCR6

**Fig 5:**
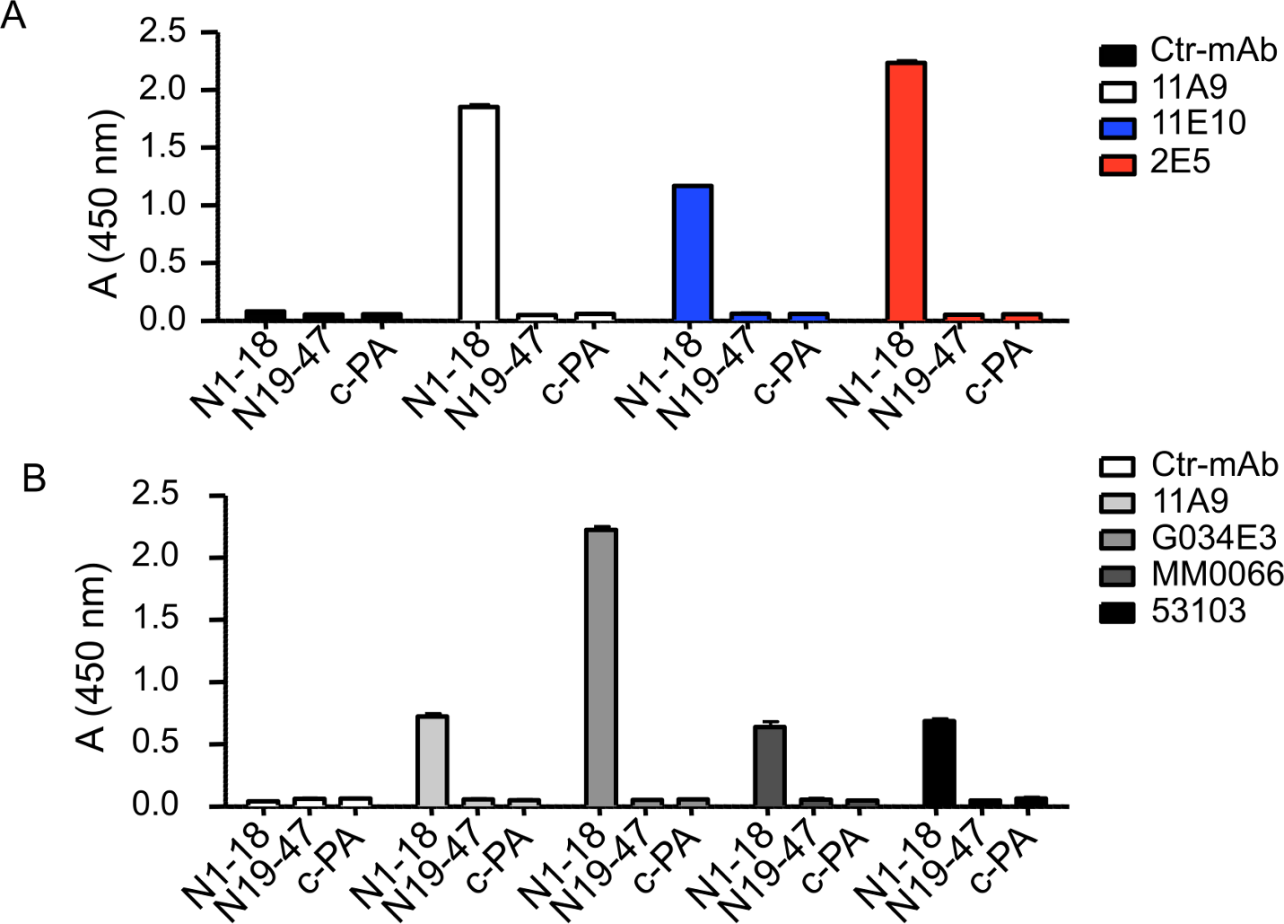
Characterization of the immunogenic region of huCCR6. The reactivity of the anti-huCCR6 mAbs **(A)** 2E5, 11E10 and 11A9 or **(B)** 53103, MM0066, G034E3 and 11A9 was compared to those of a control mAb (Ctr-mAb) by ELISA using plate-adsorbed N1-18, N19-47 and c-PA peptides. One representative analysis of three independent experiments carried out in duplicate.

Both mAbs 2E5 and 11E10 bind human CCR6 at the cell membrane as shown by flow cytometry (Fig 4A) pointing to their interaction with an extracellular domain. However, their inability to neutralize CCL20 binding and signaling (Fig 4B–4D) demonstrates that they bind a non-neutralizing region. The N-terminal part and the ECL-2 of huCCR6 are the major domains exposed outside the cell membrane with a 47 and 31 amino acid long sequence, respectively. 2E5 and 11E10 reactivity against the N-terminal domain (peptide N1-18 and N19-47) and the ECL-2 (peptide cPA) was tested by ELISA. Both mAbs showed a high level of binding with N1-18, but not with N19-47, nor cPA, similar to the mAb 11A9 (Fig 5A), generated against the N-terminal domain. Based on the finding that both 2E5 and 11E10, although harboring different variable domains, recognize the same region on huCCR6, it was hypothesized that all mAbs generated in the mouse with cells expressing native huCCR6, are specific for the N1-18 region.

**Fig 6:**
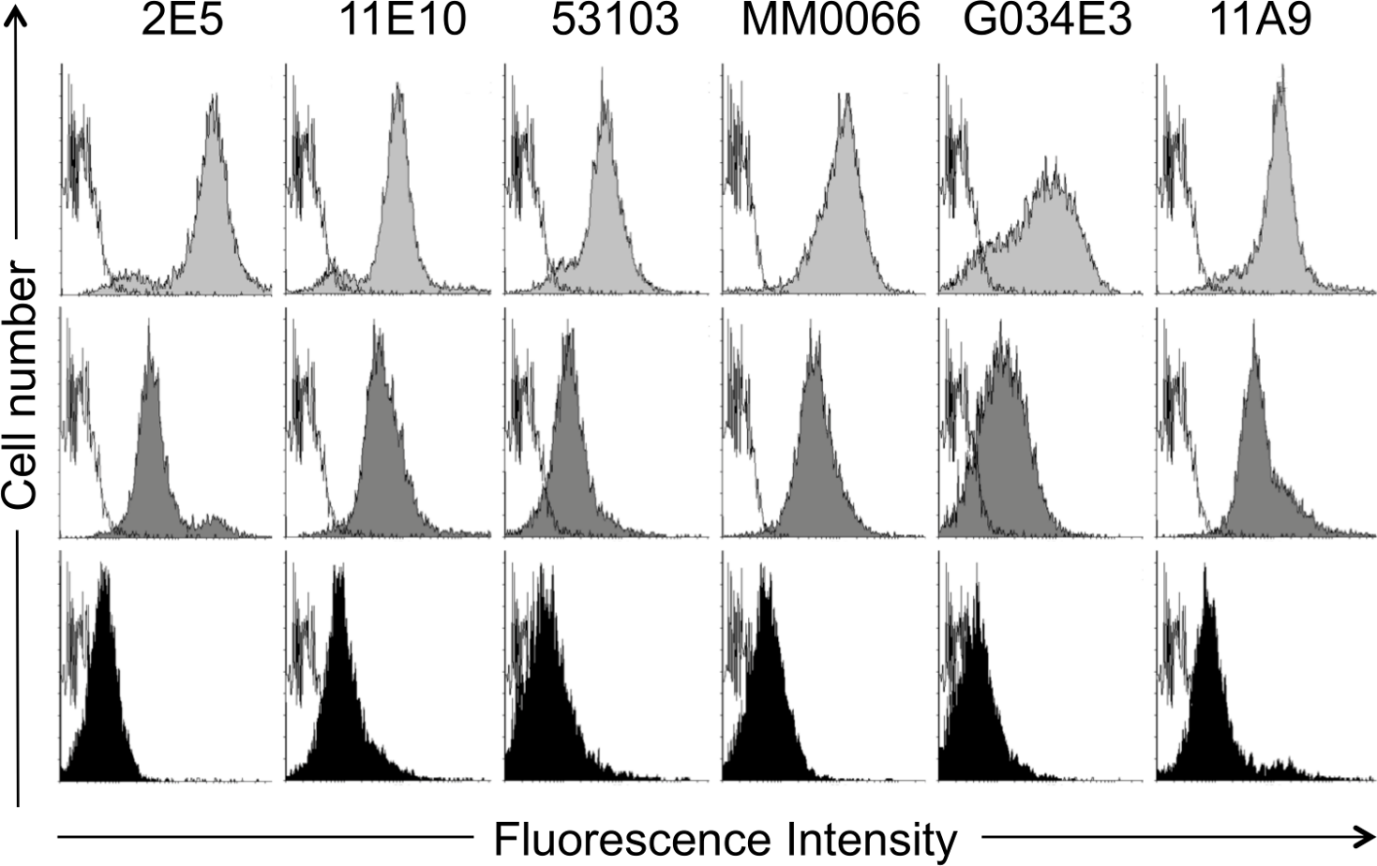
Competition by mAbs for binding to huCCR6. Binding of anti-huCCR6 mAbs (1μg/mL) to huCCR6-CHO cells, in the absence (light grey histograms) or the presence of N1-18 peptide, at the concentration of 0.1 μg/mL (dark grey histograms) or 1 μg/mL (black histogram) was determined by flow cytometry. The *x-axis* represents fluorescence (four-decade log scale), and the *y-axis* represents the relative cell number. Histograms of cells stained with a control mAb (white histograms, nearest the *y-axis*) are superimposed over histograms of cells stained with the indicated anti-huCCR6 mAbs. One representative analysis from three independent experiments is shown. The histogram overlay were performed using Flowing Software vs 2.5.

Indeed, among all the available mAbs generated in this manner, irrespective of the nature of the huCCR6-expressing transfectant cells used as the immunogen (S1 Table), each was found to specifically bind the N1-18, but not the N19-47 sequence, neither ECL-2 (Fig 5B). The linear N1-18 peptide is therefore predicted to be the likely antibody-binding site on the native receptor. This finding was confirmed by the capacity of the N1-18 peptide to displace, in a dose-dependent manner, the binding of each mAb to huCCR6-CHO cells (Fig 6). These results unambiguously demonstrate that N1-18 is the one and only region on native huCCR6 that is recognized by these mouse-derived mAbs.

## Discussion

The CCR6 chemokine receptor is selectively expressed on leukocytes populations that are involved in the pathogenesis of various chronic inflammatory diseases which is, in part, determined by the capacity of these cells to be recruited to inflamed tissue. Because of the pivotal role of CCR6 in this trafficking process, this chemokine receptor might constitute a prime target for the development of novel immunotherapeutic treatment for such diseases.

CCR6, as most 7-transmembrane domain receptors, contains relatively few extracellular residues and therefore has only a limited number of immunogenic sequences in the N-terminal portion and the three extracellular loops. Its particular nature renders it impossible to generate soluble recombinant proteins or native protein complexes for immunization purposes without losing its conformational structure that is associated with functional activity. Moreover, unlike other chemokine receptors, CCR6 is not easily amenable to the inhibitory effects of small molecules [45] and therefore other therapeutic approaches, in particular the development of neutralizing Abs, have to be explored. The present study reports various unsuccessful strategies to generate such a function-blocking mAb. Although not mentioned in the result section, it is of note that in addition to immunization with CCR6-derived peptides and the huCCR6-expressing NIH-3T3 transfectant, other methods have been explored, including immunization of Balb/C mice with native CCR6-expressing Th17 cells and the use of *in vivo* huCCR6 cDNA immunization and electroporation. These techniques have also been applied using CCR6-deficient mice, as well as mice on a C57/BL6 background, but did not yield satisfactory results. The obtention of the few mAbs specific for the huCCR6 using the hybridoma technique by us and others has involved the screening of several thousands of culture supernatants which suggests that this chemokine receptor is a rather weak immunogen in the mouse. Moreover, the results of the present study unequivocally lead to the conclusion that any mAb specific for native huCCR6, generated in the mouse, will interact with the N1-18 domain of this receptor. However, given the non-neutralizing activity of such mAbs, determination of the miminal epitope(s) that they recognize within this region does not provide additional and useful information. As to the question why these mAbs do not possess neutralizing activity, it is worth mentioning that a general model for a two-site mechanism has been proposed to describe the binding of chemokines with their cognate receptors [46]. The specific recognition is initiated by the interaction between the N-loop of the chemokine and the N-terminal domain of the chemokine receptor (site 1), followed by the engagement of the flexible N-terminal domain of the chemokine with the transmembrane domain of the chemokine receptor (site 2). Binding of mAbs to the N1-18 domain inhibits neither CCL20 binding nor receptor signaling, suggesting that this part of huCCR6 is not engaged in the recognition site of its chemokine ligand.

The molecular mechanism that is responsible for this bias in the immune response of the mouse against huCCR6 is unclear at present. It can be hypothesized that the entire N-terminal domain, as well as the ECL-2, are less accessible as a result of the conformation of huCCR6 at the surface of the cells used as an immunogen. In this respect, it has to be noted that the N-terminus of mouse CCR6 lacks the N1-5 and N19-21 residues, as compared to its human counterpart [47], resulting in a degree of homology of only 46% between the respective N-terminal portions of these chemokine receptors. This species difference is likely to result in an altered configuration, and thus a different presentation, of the molecule that might explain the observed unilateral immune response. This notion is underscored by our finding that immunization of mice with the N19-47 peptide resulted in the generation of mAbs that recognize the immunizing peptide but not native huCCR6. These results could be explained by the existence of a putative glycosylation site at residue N23 of huCCR6 that might interfere with the binding of these mouse Abs. Nonetheless, polyclonal Abs generated in goat with the synthetic peptide 18–46 reportedly react in a huCCR6-dependent manner on tissue sections [48, 49], however no information about their possible neutralizing activity and binding to the native molecule is available. In the same vein, none of the sera of mice immunized, in the present study, with peptides specific for the ECL-2 of huCCR6 reacted with native huCCR6. In addition, other huCCR6 polyclonal Abs generated against the same ECL-2-derived peptide in rabbits (LS-A356 and TA316610 Abs from LSbio and Origene, respectively) react with huCCR6 exclusively on tissue sections. These observations indicate that both sequences seem to be accessible only following denaturation of huCCR6 caused by fixation of tissue, raising the possibility that they might be masked on the native receptor by the outermost N-terminal part of the molecule. This interpretation could also explain the impediment to select small molecules to antagonize this receptor [45]. Although the company ChemoCentryx has presented promising results in preclinical models of arthritis and psoriasis, using a selective small molecule (CCX9664), these have not yet been associated with a scientific publication or beneficial results from clinical trials.

Taken together, the data from the present study indicate that the mouse humoral response against native huCCR6 is invariably directed at its N1-18 domain. However, mAbs derived from hyperimmune mice have no neutralizing activity, which leads to the postulate that this species is not suitable to develop this type of therapeutic tools to interfere with huCCR6-ligand interactions. It is of note that two recents patents described the isolation of neutralizing anti-huCCR6 antibodies, one from phage display human antibody libraries selected against huCCR6-presenting proteoliposomes [50], and the other from SD rats immunized with huCCR6-CHO cells [51]. As only rare function-blocking anti-huCCR6 antibodies have been described in other species, future attempts to generate such tools in mice, must rely on other strategies to expose critical regions [52]. In this respect, mutation or truncation of the immunodominant epitope of the N-terminus of the receptor might display unmasked and potentially neutralizing epitopes. This strategy was, for instance, reported to successfully generate broadly neutralizing antibodies against HIV-1 by removing glycans on the envelope glycoproteins [53, 54].

An alternative to the use of function-neutralizing mAbs directed at huCCR6 might be the engineering of chimerized or humanized mAbs based on the variable domains of available non-blocking mouse mAbs. For this type of mAbs, the human Fc portion will serve as a functional domain to delete CCR6-expressing cells by antibody-dependent cell-mediated cytotoxicity and/or complement-dependant cytotoxicity. Specific deletion of CCR6-expressing cells for the treatment of inflammatory disease might be a reasonable approach which is underscored by the observation that CCR6-deficient mice do not show developmental anomalies, neither of organs or tissue, nor of major leukocyte populations [4]. One specific alteration in CCR6-deficient mice is the generation of a defective secondary humoral immune reponse, most likely due to the disruption of the normal CCL20-dependent anatomic distribution in the spleen, although primary humoral responses, as well as the generation and maintenance of antigen-specific B cells, are normal in these animals [55]. Thus, suppression of antigen-specific B cells by targeting CCR6 does not seem to have major consequences. In fact, depletion of circulating B cells as a therapeutic strategy has already been successfully developed for the treatment of leukemia or rheumatoid arthritis by the use of Rituximab that targets the CD20 molecule at the B-cell surface [56–58]. Nevertheless, a definite *in vivo* demonstration of this concept with respect to CCR6 remains difficult, using the existing mAbs, as the latter are specific for huCCR6, without crossreactivity for other species usually used in experimental animal models of inflammatory diease.

Another strategy to block CCR6 function is the development of neutralizing mAbs directed at its chemokine ligand CCL20. Recently, a humanized anti-huCCL20 mAb has been evaluated in a phase 1 clinical trial (ClinicalTrials.gov Identifier: NCT01984047). It is of note however that CCR6 has other, non-chemokine, ligands, including at least two antimicrobial β-defensins [9, 24]. In this respect, neutralization of huCCL20 might not be enough to completely inhibit CCR6 function.

In fine, the results from the present study contribute to a better understanding of the reason as to why twenty years after the original description of CCR6, no neutralizing mouse mAbs have been reported, neither in the literature, nor commercially available and furthermore indicate that none one of the proposed strategies to raise a blocking antibody against CCR6 in the mouse is likely to be successful.

## Supporting Information

S1 Fig Serum antibody response in mice immunized with huCCR6-derived peptides. Serum antibody titers of mice, immunized with (A) c-PA or (B) N19-47 peptides was determined by ELISA using plate-adsorbed peptides, as indicated by the different colored bars. Results represent the values (mean ± SD) from sera of four mice measured in duplicate. Ctr-p: Control peptide.
(TIFF)

S2 Fig Internalization assay with the 2E5 and 11E10 mAbs. HuCCR6-CHO cells were incubated for 1 hour at 37°C, 5% CO2, in supplemented culture medium with various concentrations of mAbs and analyzed by flow cytometry assay. After plotting the data (log(agonist) vs. normalized response, GraphPad Prism software), the EC50 was 1.2 μM for 2E5 and 0.8 μM for 11E10. One representative analysis in duplicate from two independent experiments is shown.
(TIFF)

S1 Table Description of the anti-huCCR6 mAbs used in this study. (TIFF)

S2 Table Molecular characterization of the generated anti-huCCR6 mAbs. (TIFF)

## Abbreviations

ECL-2: second extracellular loop
huCCR6: human CCR6
c-PA: cyclic peptidyl arch
l-PA: linear peptidyl arch

## References

[1] Baba M, Imai T, Nishimura M, Kakizaki M, Takagi S, Hieshima K, et al Identification of CCR6, the specific receptor for a novel lymphocyte-directed CC chemokine LARC. Journal of Biological Chemistry. 1997;272(23):14893–8. 10.1074/jbc.272.23.14893 . 9169459

[2] Greaves DR, Wang W, Dairaghi DJ, Dieu MC, deSaintVis B, FranzBacon K, et al CCR6, a CC chemokine receptor that interacts with macrophage inflammatory protein 3 alpha and is highly expressed in human dendritic cells. Journal of Experimental Medicine. 1997;186(6):837–44. 10.1084/jem.186.6.837 . 9294138 PMC2199049

[3] Liao F, Rabin RL, Smith CS, Sharma G, Nutman TB, Farber JM. CC-chemokine receptor 6 is expressed on diverse memory subsets of T cells and determines responsiveness to macrophage inflammatory protein 3α. Journal of Immunology. 1999;162(1):186–94. . 9886385

[4] Cook DN, Prosser DM, Forster R, Zhang J, Kuklin NA, Abbondanzo SJ, et al CCR6 mediates dendritic cell localization, lymphocyte homeostasis, and immune responses in mucosal tissue. Immunity. 2000;12(5):495–503. 10.1016/s1074-7613(00)80201-0 . 10843382

[5] Le Borgne M, Etchart N, Goubier A, Lira SA, Sirard JC, van Rooijen N, et al Dendritic cells rapidly recruited into epithelial tissues via CCR6/CCL20 are responsible for CD8(+) T cell crosspriming in vivo. Immunity. 2006;24(2):191–201. 10.1016/j.immuni.2006.01.005 . 16473831

[6] Lechner A, Ritter U, Varona R, Marquez G, Bogdan C, Korner H. Protective immunity and delayed type hypersensitivity reaction are uncoupled in experimental Leishmania major infection of CCR6-negative mice. Microbes and Infection. 2007;9(3):291–9. 10.1016/j.micinf.2006.12.002 . 17317260

[7] Salazar-Gonzalez RM, Niess JH, Zammit DJ, Ravindran R, Srinivasan A, Maxwell JR, et al CCR6-mediated dendritic cell activation of pathogen-specific T cells in Peyer's patches. Immunity. 2006;24(5):623–32. 10.1016/j.immuni.2006.02.015 . 16713979 PMC2855652

[8] Varona R, Villares R, Carramolino L, Goya F, Zaballos A, Gutieerrez J, et al CCR6-deficient mice have impaired leukocyte homeostasis and altered contact hypersensitivity and delayed-type hypersensitivity responses. Journal of Clinical Investigation. 2001;107(6):R37–R45. 10.1172/jci11297 . 11254677 PMC208945

[9] Yang D, Chertov O, Bykovskaia N, Chen Q, Buffo MJ, Shogan J, et al β-defensins: Linking innate and adaptive immunity through dendritic and T cell CCR6. Science. 1999;286(5439):525–8. 10.1126/science.286.5439.525 . 10521347

[10] Acosta-Rodriguez EV, Rivino L, Geginat J, Jarrossay D, Gattorno M, Lanzavecchia A, et al Surface phenotype and antigenic specificity of human interleukin 17-producing T helper memory cells. Nature Immunology. 2007;8(6):639–46. 10.1038/ni1467 . 17486092

[11] Annunziato F, Cosmi L, Santarlasci V, Maggi L, Liotta F, Mazzinghi B, et al Phenotypic and functional features of human Th17 cells. Journal of Experimental Medicine. 2007;204(8):1849–61. 10.1084/jem.20070663 . 17635957 PMC2118657

[12] Singh SP, Zhang HH, Foley JF, Hedrick MN, Farber JM. Human T cells that are able to produce EL-17 express the chemokine receptor CCR6. Journal of Immunology. 2008;180(1):214–21. . 10.4049/jimmunol.180.1.214 18097022

[13] Krzysiek R, Lefevre EA, Bernard J, Foussat A, Galanaud P, Louache F, et al Regulation of CCR6 chemokine receptor expression and responsiveness to macrophage inflammatory protein-3 alpha/CCL20 in human B cells. Blood. 2000;96(7):2338–45. . 11001880

[14] Serafini N, Vosshenrich CAJ, Di Santo JP. Transcriptional regulation of innate lymphoid cell fate. Nature Reviews Immunology. 2015;15(7):415–28. 10.1038/nri3855 . 26065585

[15] Dieu MC, Vanbervliet B, Vicari A, Bridon JM, Oldham E, Ait-Yahia S, et al Selective recruitment of immature and mature dendritic cells by distinct chemokines expressed in different anatomic sites. Journal of Experimental Medicine. 1998;188(2):373–86. 10.1084/jem.188.2.373 . 9670049 PMC2212459

[16] Reboldi A, Coisne C, Baumjohann D, Benvenuto F, Bottinelli D, Lira S, et al C-C chemokine receptor 6-regulated entry of T-H-17 cells into the CNS through the choroid plexus is required for the initiation of EAE. Nature Immunology. 2009;10(5):514–23. 10.1038/ni.1716 . 19305396

[17] Liston A, Kohler RE, Townley S, Haylock-Jacobs S, Comerford I, Caon AC, et al Inhibition of CCR6 Function Reduces the Severity of Experimental Autoimmune Encephalomyelitis via Effects on the Priming Phase of the Immune Response. Journal of Immunology. 2009;182(5):3121–30. 10.4049/jimmunol.0713169 . 19234209

[18] Hirota K, Yoshitomi H, Hashimoto M, Maeda S, Teradaira S, Sugimoto N, et al Preferential recruitment of CCR6-expressing Th17 cells to inflamed joints via CCL20 in rheumatoid arthritis and its animal model. J Exp Med. 2007;204(12):2803–12. jem.20071397 [pii] 10.1084/jem.20071397 18025126 PMC2118525

[19] Kochi Y, Okada Y, Suzuki A, Ikari K, Terao C, Takahashi A, et al A regulatory variant in CCR6 is associated with rheumatoid arthritis susceptibility. Nature Genetics. 2010;42(6):515–U63. 10.1038/ng.583 . 20453841

[20] Stahl EA, Raychaudhuri S, Remmers EF, Xie G, Eyre S, Thomson BP, et al Genome-wide association study meta-analysis identifies seven new rheumatoid arthritis risk loci. Nature Genetics. 2010;42(6):508–U56. 10.1038/ng.582 . 20453842 PMC4243840

[21] Hauser SL, Waubant E, Arnold DL, Vollmer T, Antel J, Fox RJ, et al B-cell depletion with Rituximab in relapsing-remitting multiple sclerosis. New England Journal of Medicine. 2008;358(7):676–88. 10.1056/NEJMoa0706383 . 18272891

[22] Butcher EC, Picker LJ. Lymphocyte homing and homeostasis. Science. 1996;272(5258):60–6. 10.1126/science.272.5258.60 . 8600538

[23] Campbell JJ, Hedrick J, Zlotnik A, Siani MA, Thompson DA, Butcher EC. Chemokines and the arrest of lymphocytes rolling under flow conditions. Science. 1998;279(5349):381–4. 10.1126/science.279.5349.381 . 9430588

[24] Ghannam S, Dejou C, Pedretti N, Giot J-P, Dorgham K, Boukhaddaoui H, et al CCL20 and β-Defensin-2 Induce Arrest of Human Th17 Cells on Inflamed Endothelium In Vitro under Flow Conditions. Journal of Immunology. 2011;186(3):1411–20. 10.4049/jimmunol.1000597 . 21178014

[25] Pene J, Chevalier S, Preisser L, Venereau E, Guilleux M-H, Ghannam S, et al Chronically inflamed human tissues are infiltrated by highly differentiated Th17 lymphocytes. Journal of Immunology. 2008;180(11):7423–30. . 10.4049/jimmunol.180.11.7423 18490742

[26] Yssel H, Spits H. Generation and maintenance of cloned human T cell lines. Curr Protoc Immunol. 2002; Chapter 7:Unit 7 19. 10.1002/0471142735.im0719s47 . 18432890

[27] Yssel H, de Vries JE, Koken M, van Blitterswijk W, Spits H. Serum-free medium for generation and propagation of functional human cyto-toxic and helper t-cell clones. Journal of Immunological Methods. 1984;72(1):219–27. 10.1016/0022-1759(84)90450-2 . 6086760

[28] Chen TR. Insitu detection of mycoplasma contamination in cell-cultures by fluorescent hoechst-33258 stain. Experimental Cell Research. 1977;104(2):255–62. 10.1016/0014-4827(77)90089-1 . 65285

[29] Raja Z, Andre S, Piesse C, Sereno D, Nicolas P, Foulon T, et al Structure, Antimicrobial Activities and Mode of Interaction with Membranes of Bovel Phylloseptins from the Painted-Belly Leaf Frog, Phyllomedusa sauvagii. Plos One. 2013;8(8). 10.1371/journal.pone.0070782 . PMC3742671 23967105

[30] Yokoyama WM, Christensen M, Dos Santos G, Miller D, Ho J, Wu T, et al Production of monoclonal antibodies. Current protocols in immunology / edited by John E Coligan et al 2013;102:Unit 2.5.-Unit 2.5. 10.1002/0471142735.im0205s102 . 24510488

[31] Dorgham K, Dogan I, Bitton N, Parizot C, Cardona V, Debre P, et al Immunogenicity of HIV type 1 gp120 CD4 binding site phage mimotopes. Aids Research and Human Retroviruses. 2005;21(1):82–92. . 15665647 10.1089/aid.2005.21.82

[32] Brochet X, Lefranc MP, Giudicelli V. IMGT/V-QUEST: the highly customized and integrated system for IG and TR standardized V-J and V-D-J sequence analysis. Nucleic Acids Res. 2008;36(Web Server issue):W503–8. 10.1093/nar/gkn316 18503082 PMC2447746

[33] Giudicelli V, Brochet X, Lefranc MP. IMGT/V-QUEST: IMGT standardized analysis of the immunoglobulin (IG) and T cell receptor (TR) nucleotide sequences. Cold Spring Harb Protoc. 2011;2011(6):695–715. 10.1101/pdb.prot5633 . 21632778

[34] Fernandez EJ, Lolis E. Structure junction, and inhibition of chemokines. Annual Review of Pharmacology and Toxicology. 2002;42:469–99. 10.1146/annurev.pharmtox.42.091901.115838 . 11807180

[35] Ai LS, Lee SF, Chen SSL, Liao F. Molecular characterization of CCR6: Involvement of multiple domains in ligand binding and receptor signaling. Journal of Biomedical Science. 2004;11(6):818–28. 10.1159/000081829 . 15591779

[36] Ai LS, Liao F. Mutating the four extracellular cysteines in the chemokine receptor CCR6 reveals their differing roles in receptor trafficking, ligand binding, and signaling. Biochemistry. 2002;41(26):8332–41. 10.1021/bi025855y . 12081481

[37] Tan Q, Zhu Y, Li J, Chen Z, Han GW, Kufareva I, et al Structure of the CCR5 Chemokine Receptor-HIV Entry Inhibitor Maraviroc Complex. Science. 2013;341(6152):1387–90. 10.1126/science.1241475 . 24030490 PMC3819204

[38] Qin L, Kufareva I, Holden LG, Wang C, Zheng Y, Zhao C, et al Crystal structure of the chemokine receptor CXCR4 in complex with a viral chemokine. Science. 2015;347(6226):1117–22. 10.1126/science.1261064 . 25612609 PMC4362693

[39] Wu B, Chien EYT, Mol CD, Fenalti G, Liu W, Katritch V, et al Structures of the CXCR4 Chemokine GPCR with Small-Molecule and Cyclic Peptide Antagonists. Science. 2010;330(6007):1066–71. 10.1126/science.1194396 . 20929726 PMC3074590

[40] Burg JS, Ingram JR, Venkatakrishnan AJ, Jude KM, Dukkipati A, Feinberg EN, et al Structural basis for chemokine recognition and activation of a viral G protein-coupled receptor. Science. 2015;347(6226):1113–7. 10.1126/science.aaa5026 . 25745166 PMC4445376

[41] Misumi S, Endo M, Mukai R, Tachibana K, Umeda M, Honda T, et al A novel cyclic peptide immunization strategy for preventing HIV-1/AIDS infection and progression. Journal of Biological Chemistry. 2003;278(34):32335–43. 10.1074/jbc.M301209200 . 12771150

[42] Misumi S, Nakajima R, Takamune N, Shoji S. A cyclic dodecapeptide-multiple-antigen peptide conjugate from the undecapeptidyl arch (from Arg(168) to Cys(178)) of extracellular loop 2 in CCR5 as a novel human immunodeficiency virus type 1 vaccine. Journal of Virology. 2001;75(23):11614–20. 10.1128/jvi.75.23.11614-11620.2001 . 11689643 PMC114748

[43] Carramolino L, Kremer L, Goya I, Varona R, Buesa JM, Gutierrez J, et al Down-regulation of the beta-chemokine receptor CCR6 in dendritic cells mediated by TNF-alpha and IL-4. J Leukoc Biol. 1999;66(5):837–44. . 10577517 10.1002/jlb.66.5.837

[44] Hornbeck P, Fleisher TA, Papadopoulos NM. Isotype determination of antibodies. Current protocols in immunology / edited by John E Coligan [et al]. 2001;Chapter 2:Unit 2.-Unit. 10.1002/0471142735.im0202s01 . 18432767

[45] Mackay CR. Moving targets: cell migration inhibitors as new anti-inflammatory therapies. Nature Immunology. 2008;9(9):988–98. 10.1038/ni.f.210 . 18711436

[46] Scholten DJ, Canals M, Maussang D, Roumen L, Smit MJ, Wijtmans M, et al Pharmacological modulation of chemokine receptor function. British Journal of Pharmacology. 2012;165(6):1617–43. 10.1111/j.1476-5381.2011.01551.x . 21699506 PMC3372818

[47] Varona R, Zaballos A, Gutierrez J, Martin P, Roncal F, Albar JP, et al Molecular cloning, functional characterization and mRNA expression analysis of the murine chemokine receptor CCR6 and its specific ligand MIP-3 alpha. Febs Letters. 1998;440(1–2):188–94. 10.1016/s0014-5793(98)01450-1 . 9862452

[48] Salim SaY, Silva MA, Keita AV, Larsson M, Andersson P, Magnusson K-E, et al CD83(+)CCR7(-) Dendritic Cells Accumulate in the Subepithelial Dome and Internalize Translocated Escherichia coli HB101 in the Peyer's Patches of Heal Crohn's Disease. American Journal of Pathology. 2009;174(1):82–90. 10.2353/ajpath.2009.080273 . 19095953 PMC2631321

[49] Minamiya Y, Saito H, Takahashi N, Ito M, Toda H, Ono T, et al Expression of the chemokine receptor CCR6 correlates with a favorable prognosis in patients with adenocarcinoma of the lung. Tumor Biology. 2011;32(1):197–202. 10.1007/s13277-010-0113-x . 20872189

[50] Kim E, Vasilyeva V, Abbasova S, Ivanova E, Ovchinnikova E, Ulitin A, et al Human monoclonal antibodies against human chemokine receptor ccr6: Google Patents; 2013 2013.

[51] Isoda Y, Koyama M, Kanda Y, Yamano K. Anticorps anti-ccr6 humain: Google Patents; 2013 2013.

[52] Alex Klarenbeek DM, Christophe Blanchetot, Michael Saunders, Sebastian van der Woning, Martine Smit, Hans de Haard, Erik Hofman. Targeting chemokines and chemokine receptors with antibodies. Drug discovery today Technologies. 2012;9(4):e227–314. 10.1016/j.ddtec.2012.05.003 . 24063738

[53] Ma B-J, Alam M, Go EP, Lu X, Desaire H, Tomaras GD, et al Envelope Deglycosylation Enhances Antigenicity of HIV-1 gp41 Epitopes for Both Broad Neutralizing Antibodies and Their Unmutated Ancestor Antibodies. Plos Pathogens. 2011;7(9). 10.1371/journal.ppat.1002200 . PMC3164629 21909262

[54] Sok D, Doores KJ, Briney B, Le KM, Saye-Francisco KL, Ramos A, et al Promiscuous Glycan Site Recognition by Antibodies to the High-Mannose Patch of gp120 Broadens Neutralization of HIV. Science Translational Medicine. 2014;6(236). 10.1126/scitranslmed.3008104 . PMC4095976 24828077

[55] Elgueta R, Marks E, Nowak E, Menezes S, Benson M, Raman VS, et al CCR6-Dependent Positioning of Memory B Cells Is Essential for Their Ability To Mount a Recall Response to Antigen. Journal of Immunology. 2015;194(2):505–13. 10.4049/jimmunol.1401553 . PMC4282958 25505290

[56] Rubbert-Roth A, Tak PP, Zerbini C, Tremblay J-L, Carreno L, Armstrong G, et al Efficacy and safety of various repeat treatment dosing regimens of rituximab in patients with active rheumatoid arthritis: results of a Phase III randomized study (MIRROR). Rheumatology. 2010;49(9):1683–93. 10.1093/rheumatology/keq116 . 20463186 PMC2919195

[57] Dufour A, Palermo G, Zellmeier E, Mellert G, Duchateau-Nguyen G, Schneider S, et al Inactivation of TP53 correlates with disease progression and low miR-34a expression in previously treated chronic lymphocytic leukemia patients. Blood. 2013;121(18):3650–7. 10.1182/blood-2012-10-458695 . 23525797

[58] Weisser M, Yeh R-F, Duchateau-Nguyen G, Palermo G, Tri Quang N, Shi X, et al PTK2 expression and immunochemotherapy outcome in chronic lymphocytic leukemia. Blood. 2014;124(3):420–5. 10.1182/blood-2013-12-538975 . 24916506

