# Supplementary figures and images for: Identification of the Single Immunodominant Region of the Native Human CC Chemokine Receptor 6 Recognized by Mouse Monoclonal Antibodies
Source: PLoS One. 2016 Jun 23;11(6):e0157740. doi: 10.1371/journal.pone.0157740 (PMC4919008; doi:10.1371/journal.pone.0157740)

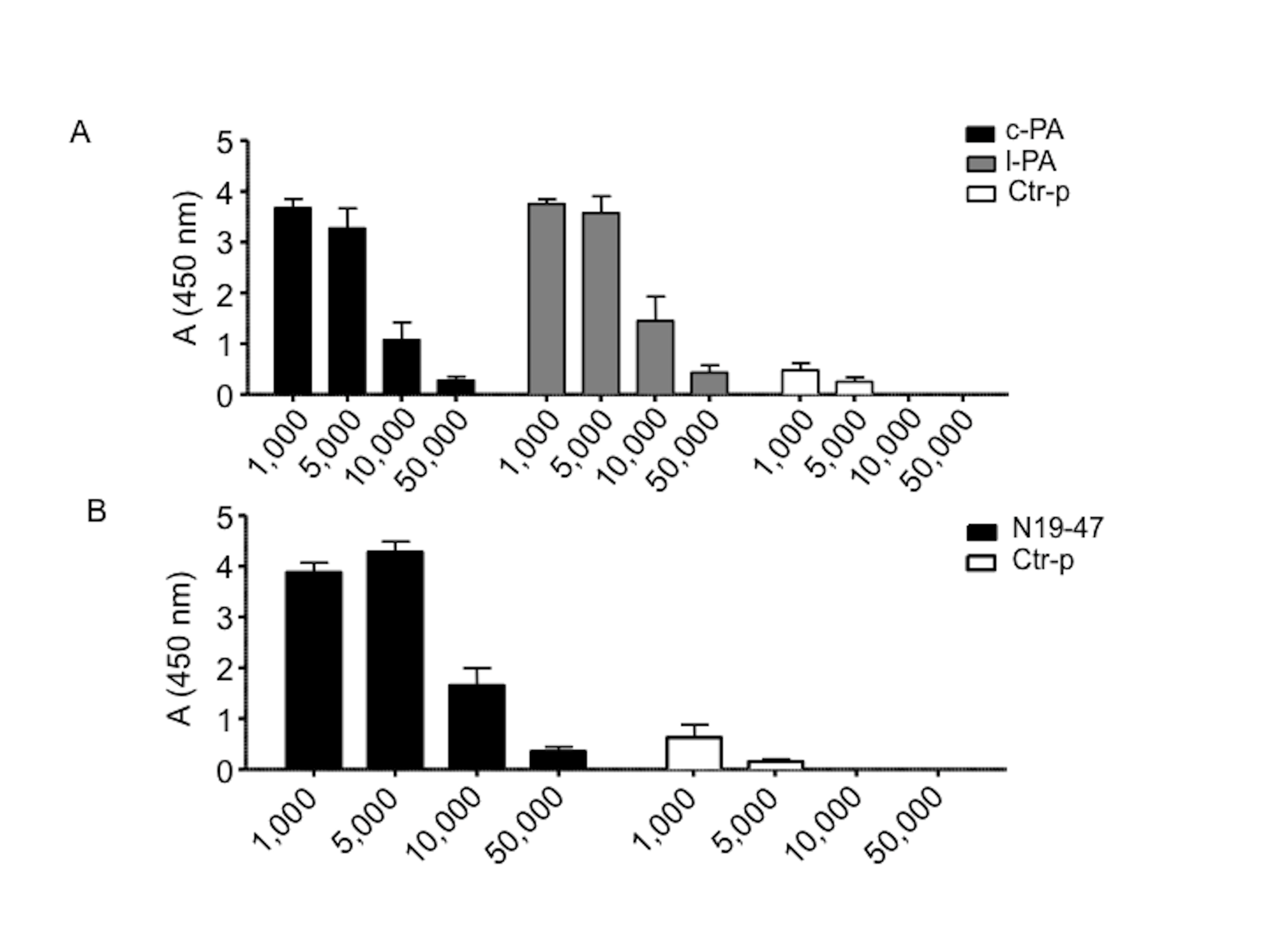

Supplement: S1 Fig — Serum antibody titers of mice, immunized with (A) c-PA or (B) N19-47 peptides was determined by ELISA using plate-adsorbed peptides, as indicated by the different colored bars. Results represent the values (mean ± SD) from sera of four mice measured in duplicate. Ctr-p: Control peptide. (TIFF) [file pone.0157740.s001.tiff]

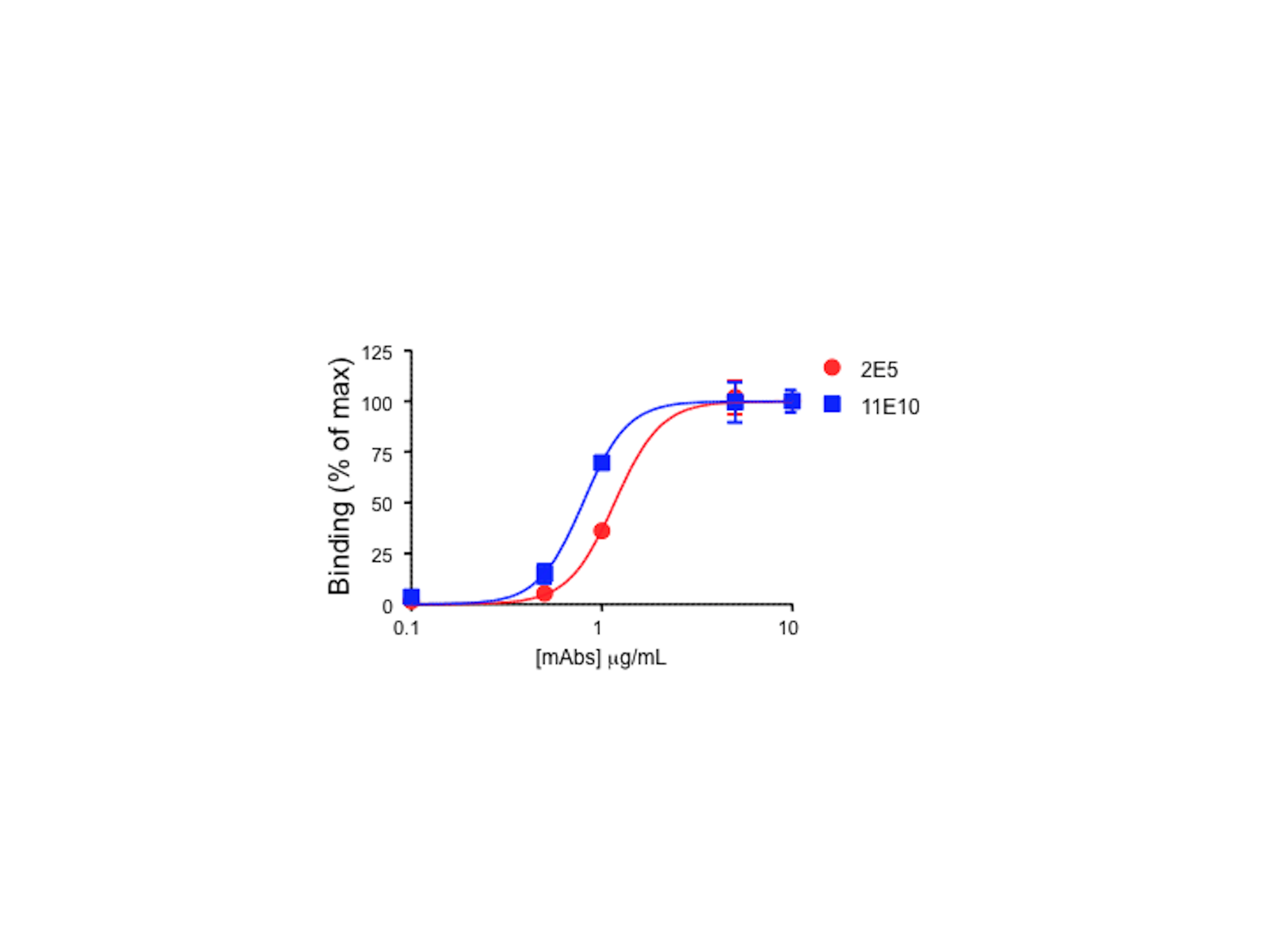

Supplement: S2 Fig — HuCCR6-CHO cells were incubated for 1 hour at 37°C, 5% CO2, in supplemented culture medium with various concentrations of mAbs and analyzed by flow cytometry assay. After plotting the data (log(agonist) vs. normalized response, GraphPad Prism software), the EC50 was 1.2 μM for 2E5 and 0.8 μM for 11E10. One representative analysis in duplicate from two independent experiments is shown. (TIFF) [file pone.0157740.s002.tiff]

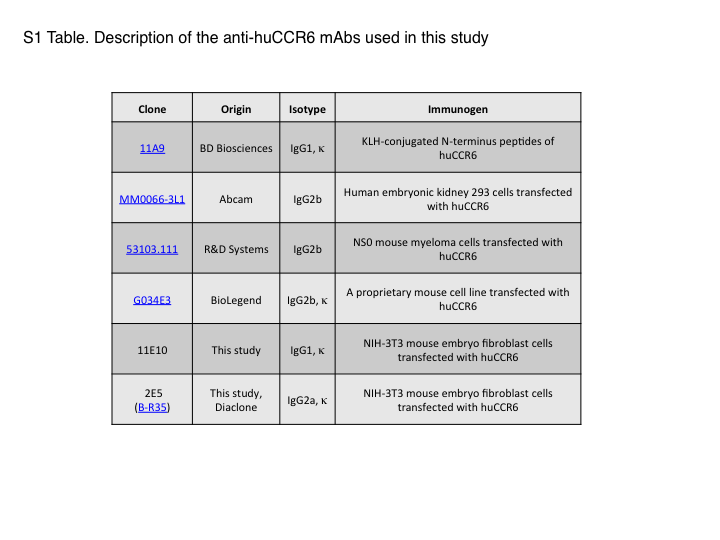

Supplement: S1 Table — (TIFF) [file pone.0157740.s003.tiff]

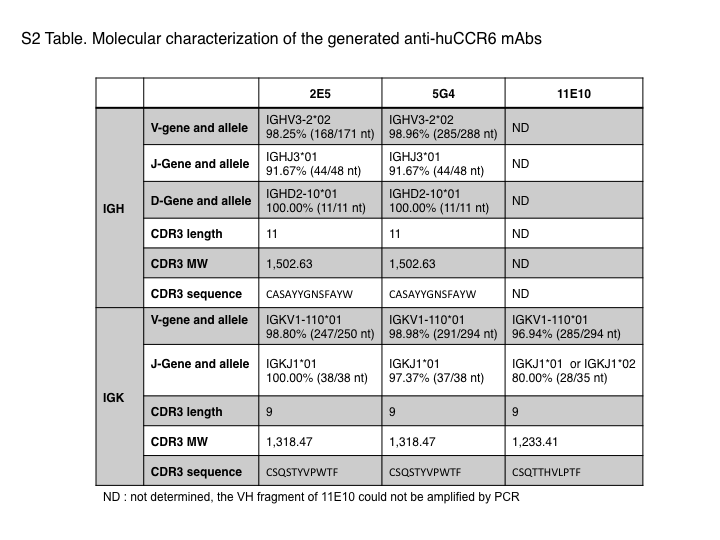

Supplement: S2 Table — (TIFF) [file pone.0157740.s004.tiff]
